# Establishment and inheritance of minichromosomes from Arabidopsis haploid induction

**DOI:** 10.1007/s00412-023-00788-5

**Published:** 2023-03-25

**Authors:** Ek Han Tan, Benny Ordoñez, Tejas Thondehaalmath, Danelle K. Seymour, Julin N. Maloof, Ravi Maruthachalam, Luca Comai

**Affiliations:** 1 Plant Biology and Genome Center, University of California, 1 Shields Avenue, DavisDavis, CA 95616 USA; 2 School of Biology and Ecology, University of Maine, Presque Isle, Orono, ME 04469 USA; 3 School of Biology, Indian Institute of Science Education and Research (IISER), Thiruvananthapuram, Vithura, Kerala 695551 India; 4 Department of Botany and Plant Sciences, University of California, Riverside, CA USA

**Keywords:** Genome instability, Genome elimination, Chromosome fragmentation, DNA deletion, DNA vector, Centromere

## Abstract

Minichromosomes are small, sometimes circular, rearranged chromosomes consisting of one centromere and short chromosomal arms formed by treatments that break DNA, including plant transformation. Minichromosomes have the potential to serve as vectors to quickly move valuable genes across a wide range of germplasm, including into adapted crop varieties. To realize this potential, minichromosomes must be reliably generated, easily manipulated, and stably inherited. Here we show a reliable method for minichromosome formation in haploids resulting from CENH3-mediated genome elimination, a process that generates genome instability and karyotypic novelty specifically on one parental genome. First, we identified 2 out of 260 haploids, each containing a single-copy minichromosome originating from centromeric regions of chromosomes 1 and 3, respectively. The chromosome 1 minichromosome we characterized did not pair at meiosis but displayed consistent transmission over nine selfing generations. Next, we demonstrated that CENH3-based haploid induction can produce minichromosomes in a targeted manner. Haploid inducers carrying a selectable pericentromeric marker were used to isolate additional chromosome-specific minichromosomes, which occurred in 3 out of 163 haploids. Our findings document the formation of heritable, rearranged chromosomes, and we provide a method for convenient minichromosome production.

## Introduction

Minichromosomes are small chromosome-like structures that arise from spontaneous or induced deletion derivatives of either a standard chromosome or a supernumerary B-chromosome. They consist of one centromere (rarely more (Murata 2014)), one or more origins of replication, and can be either linear with two telomere-capped ends or circular with covalently joined arms to form a ring minichromosome. Aneuploidy, or the presence of additional chromosomes, results in an imbalance in the dosage of genes carried by the extra chromosome and is often deleterious (Birchler and Veitia 2012). Minichromosomes carry fewer genes, with any remaining perturbation in gene dosage more likely to be tolerated in aneuploids. As a result, minichromosomes can provide a platform for genome engineering through deployment of specialized chromosomal vectors.

The stable inheritance of minichromosomes is essential for directed genetic manipulation, but their reduced size presents a set of challenges for transmission. Frequently, both linear and circular minichromosomes display impaired mitotic and meiotic transmission as well as failure of sister chromatid cohesion and meiotic pairing (McClintock 1941; Schwartz 1958; Murata 2014; Birchler 2015). During mitotic divisions, an uneven number of crossovers between circular sister chromatids forms a larger dicentric circle (McClintock 1938). The two opposing centromeres result in bridge-breakage-fusion cycles and recurring deletions and duplications. Analysis of linear B-A minichromosomes in maize showed that meiotic pairing was often but not always affected. The decreasing size of minichromosomes increased the probability of precocious sister chromatid separation by altering the cohesive properties of centromeres (Han et al. 2007). In summary, the use of minichromosomes in biotechnology will benefit from selection systems in gametes, seeds, or seedlings (Han et al. 2007). A better understanding of their structural stability will be useful to tailor specific uses. Indeed, instability could be leveraged for temporary delivery of certain transgene products, such as for gene editing (Birchler et al. 2016).

In plants, uniparental genome elimination leads to large-scale missegregation. Genome elimination can ensue when a haploid inducer, a strain expressing an altered CENH3, is pollinated by a wild-type strain (Ravi and Chan 2010). The centromeres of chromosomes contributed by the haploid inducer lack a sufficient density of CENH3 chromatin and fail to recruit centromere and kinetochore factors during mitotic cell cycles in the early embryo. The epigenetic deficit of these centromeres in comparison to the wild-type ones results in missegregation of the affected chromosomes, formation of micronuclei, and frequent loss of haploid inducer chromosomes (Marimuthu et al. 2021). Instability of the affected chromosomes can produce novel chromosomes that are highly rearranged by chromoanagenesis (Tan et al. 2015). Chromoanagenesis is an umbrella term defining the outcome of catastrophic event(s) driven by DNA double strand breaks (DSBs) and leading to rearrangement of DNA fragments in random order and orientation to form complex derivative chromosomes (Guo et al. 2023). Chromosomes segregated into defective cellular compartments, such as micronuclei, can undergo fragmentation followed by random ligation via non-homologous end joining (NHEJ) and reconstitution of remodeled chromosomes.

During genomic analysis of progeny plants resulting from a haploid induction cross, we found evidence of minichromosomes resulting from the reduction of haploid inducer chromosomes. We present here the characterization of these events. In 1 to 2% of phenotypically normal haploids, we found the presence of minichromosomes spanning the centromere and a small portion of the adjacent pericentromeric region. We show that using haploid inducers that carry a selectable transgene near a centromere, we can isolate minichromosomes encompassing that centromere. We describe the cytological, molecular, and genetic properties of selected minichromosomes, demonstrating relative stability consistent with that reported in previous minichromosome studies. Frequent minichromosome formation in these crosses has interesting evolutionary and biotechnological implications, discussed here.

## Materials and Methods

### Plant material and plant growth

**Fig. 1:**
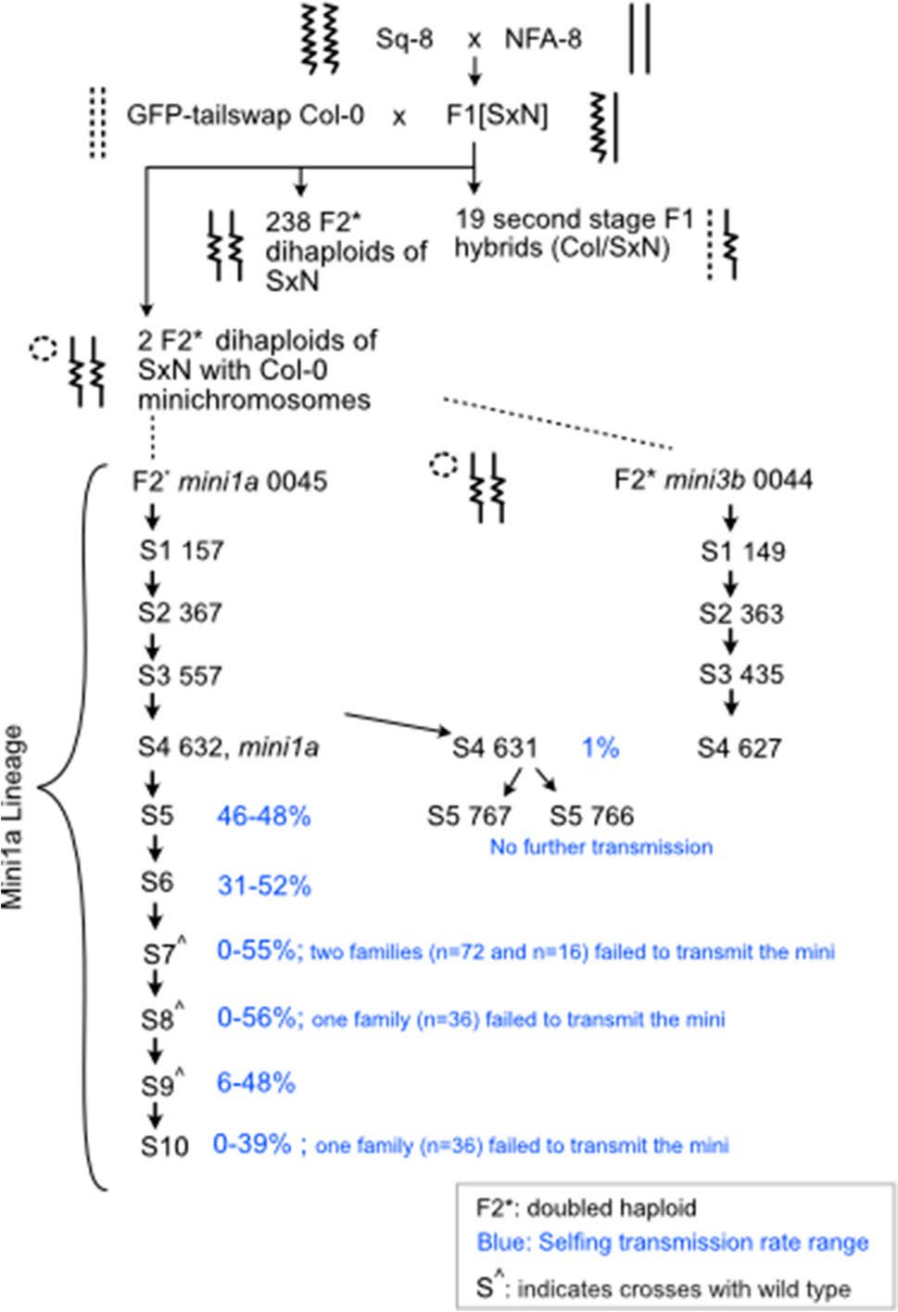
Pedigree describing the production and characterization of minichromosomes from centromere-mediated genome elimination. Transmission rates are summarized for the advanced lineage of *mini1a* lines. Detailed rates are provided in Tables 2 and 3, including outcrossing rates to wild-type L*er* *gl1* measured for generations S7–9. Smooth, hatched, and squiggly lines depict the genome’s origin. Second-stage F1s are the expected result of failed Col-0 genome elimination (Seymour et al. 2012)

The minichromosome lines were identified in the progeny of a CENH3-based genome elimination cross using the F1 hybrid of Sq-8 (CS22601)/NFA-8 (CS22598)) ecotypes in Seymour et al. (2012) and the GFP-tailswap strain in the Columbia (Col-0) background (Ravi and Chan 2010). Among the resulting doubled haploids, genotyping by sequencing analysis indicated that two individuals contained short segments of the haploid inducer (Col-0) DNA spanning the centromere on chromosomes 1 and 3 (Seymour et al. 2012). These lines from the population are DHR194 and DHR128, hereafter *mini1a* and *mini3b*, respectively (Fig. 1).

### Generational stability and meiotic stability of minichromosomes

We selected *mini1a* for further studies due to our preliminary observation that *mini1a* could be transmitted at ~ 40% frequency in the S1 to S4 generations. To analyze the meiotic behavior of *mini1a*, we conducted selfing and crosses in six consecutive generations using *mini1a* lines. Each *mini 1a*-carrying individual was allowed to self and subsequent selfed seeds were genotyped for the presence of *mini1a* using the Mini1a PCR (Table 1). We performed reciprocal crosses between wild-type Landsberg *erecta* and *mini1a-*carrying individuals. When the siliques matured, all F1 seeds from dehiscing siliques were collected. The Mini1a PCR was also used to determine the segregation of *mini1a* in the F1 progenies. To calculate the expected parental transmission rate upon selfing, we used the following formula: 1 − (1 − 0.125) * (1 − 0.107) = 0.218, where 0.125 and 0.107 are the outcrossing transmission rates for female and male gametes, respectively.

**Table 1:** Primer sequences used for *mini1a* and *mini3b* detection

Name	Sequence (5′–3′)
SNP1:13,405,811 F SspI	TGAGAACTCACTAGATGCGAGGA
SNP1:13,405,811 R SspI	GCTAAGCACTCAACTAACTTCTGTCAG
SNP3:16,409,164 F BstAPI	CCTCTCTTGGAGCAGTGATTGGAG
SNP3:16,409,164 R BstAPI	GCAAGAATTCAAGAGTCCTTTGTGGTTTG
Mini1a Fwd17.8 M	CTAGTGATTTAACGTATTGACCA
Mini1a Rev13.3 M	GAGATGTACCTTGTATCTTGAA

### Cytological characterization of minichromosome lines

To investigate the behavior of the *mini1a* in meiosis, young flower buds at different stages were collected and fixed in Carnoy’s fixative containing 60% ethanol, 30% chloroform, and 10% glacial acetic acid. Meiotic spreads were performed as described previously (Ravi et al. 2011), and male meiocyte stages were imaged using DAPI staining.

### Sequencing of minichromosome lines

Genomic analyses were performed on DNA extracted from F2 doubled haploid lines of *mini1a* and *mini3b* and F1 haploid lines from *mini4a*, *mini4b*, and *mini4c*. DNA was extracted from leaves using the Nucleon PhytoPure kit (GE/Cytiva RPN8510). For each sample, 1.5 μg of DNA was sheared on the Covaris E220 to 300–400 bp and used to construct PCR-free Illumina libraries using the NEB Next library construction kit. Samples were pooled and sequenced using HiSeq 2000 as 100-bp paired-end reads. Raw sequencing reads from this project can be accessed from BioProject ID PRJNA826082.

### Bioinformatic analyses

Dosage analyses to detect minichromosomes were performed using the bin-by-sam method after quality measures were satisfied (Henry et al. 2015). The breakpoint junction for *mini1a* was identified using breakpoint junction reconstruction methods described previously (Tan et al. 2015), and paired reads containing mismatched distances were assembled using targeted functions of the PRICE assembler (Ruby et al. 2013). PCR primers were designed from contigs obtained from PRICE and confirmed using Sanger sequencing (Table 1).

### Genotyping assays

SNP analyses for *mini1a* and *mini3b* were performed using CAPS markers as well as junction PCR for *mini1a*. Oligo sequences used are in Table 1 below.

### Cycling and digestion parameters for CAPS assay and Mini1a PCR

PCR for cleaved amplified polymorphic sequence (CAPS) assays were performed using 15 μL reaction volumes and recommended primer pair concentrations using GoTaq Green Mastermix (Promega M7122) and 55 °C annealing temperatures for 40 cycles. After PCR, a 5 μL digestion mix containing 0.1 μL restriction enzyme SspI (for SNP1:13,405,811) or BstAPI (for SNP3:16,409,164), 1 μL CutSmart Buffer (NEB), and 3.9 uL nuclease-free water was added to final PCR and digested overnight at 37 °C. The resulting digestion products were visualized on a 1% TAE gel containing ethidium bromide the next day. For SNP1:13,405,811, SspI cleaves the Col-0 SNP resulting in 216 and 410 bp products and an undigested band of 626 bp is indicative of the NFA1/SQ-1 SNP. For SNP3:16,409,164, BstAPI cleaves the NFA1/SQ-1 SNP resulting in 127 and 322 bp products and an undigested band of 449 bp indicative of the Col-0 SNP.

Mini1a PCR assays were also performed using GoTaq Green Mastermix (Promega M7122) and 55 ℃ annealing temperatures for 40 cycles. A band of 474 bp is present if *mini1a* is present and can be visualized on a 1% TAE gel containing ethidium bromide.

### Selectable minichromosome from GFP-tailswap #11 haploid inducer

A cross of T-DNA insertion line from the Syngenta Arabidopsis Insertion Library (SAIL) collection (Sessions et al. 2002), SAIL_618_H09, with left border position located on 3,942,727–3,943,148 on the top pericentromeric region of Chr4, was made with *cenh3-1*/*CENH3* GFP-tailswap #11 line. The SAIL T-DNA collection contains the *Bar* selectable marker, which confers resistance to the Basta (glufosinate ammonium) herbicide. Subsequent haploid inducer was identified in the F2 generation carrying the SAIL_618_H09 T-DNA, and *cenh3-1*/*cenh3-1* GFP-tailswap #11 was used as a haploid inducer using L*er* *gl1* as the pollen parent. F1 seeds from these crosses were surface sterilized and sown on MS plates, and L*er gl1* haploids were transferred to soil. In total, 2 weeks after transplanting, selection on haploids was performed using a spray solution containing 0.02% Basta. Out of 163 L*er gl1* haploids, three phenotypically wild-type Basta-resistant L*er gl1* lines were identified, and sequencing analyses were performed to characterize these three lines.

## Results

### Discovery of minichromosomes

**Fig. 2:**
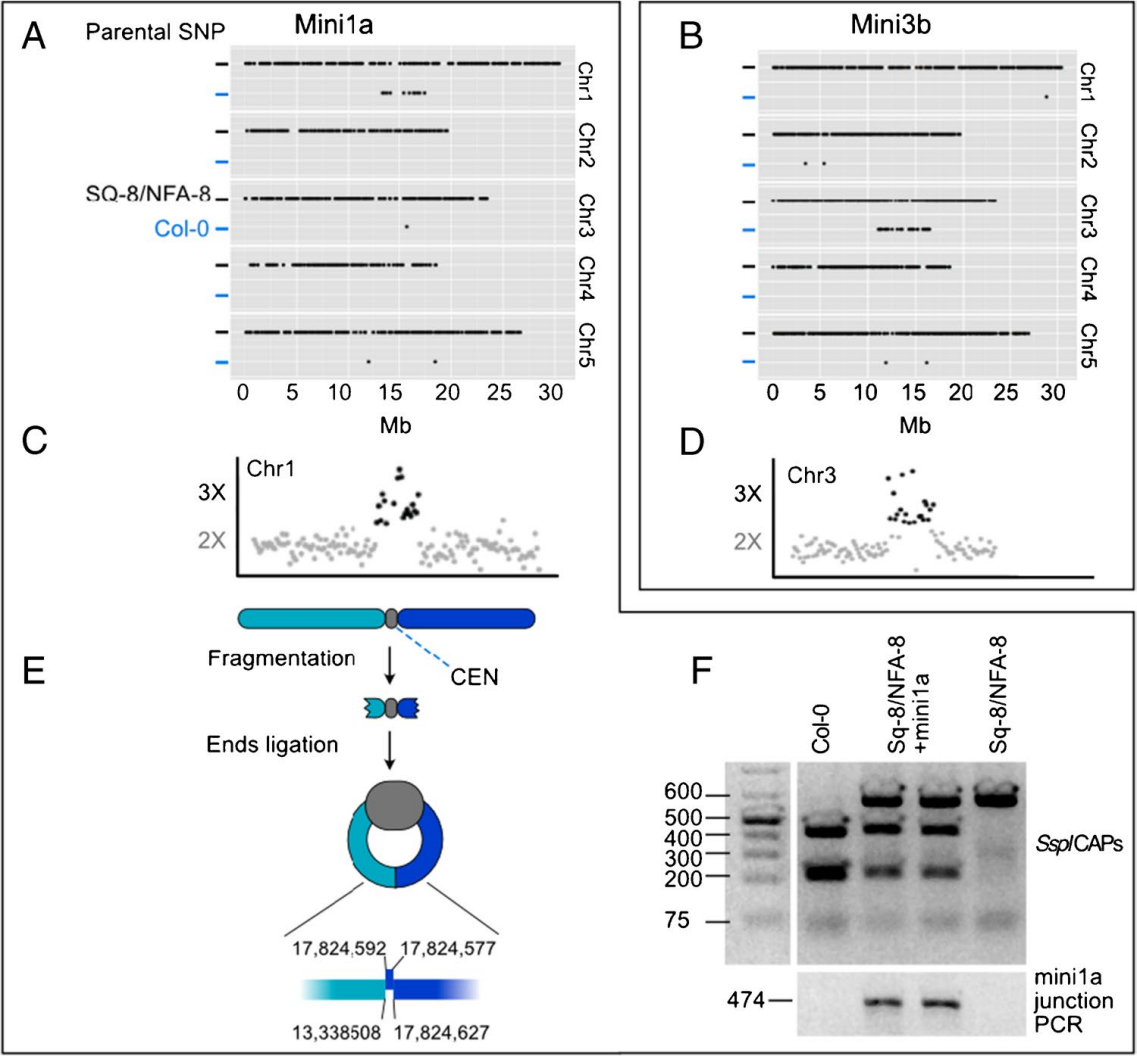
Detection and analysis of minichromosomes. The presence of *mini1a* and *mini3b* minichromosomes derived from the GFP-tailswap haploid inducer (Col-0 ecotype) in haploids induced from a Nfa8/Sq8 F1 hybrid was confirmed by genomic analysis. **A**, **B** Detection of Col-0 SNPs on chromosome 1 (Chr1) and chromosome 3 (Chr3) of haploid individuals. **C**, **D** Dosage plot of F2 doubled haploid lines containing *mini1a* and *mini3b*. **E** Origin, inferred structure, and breakpoint junction of *mini1a* at circularization site. **F** A cleaved amplified polymorphic sequence (CAPS) assay using the restriction enzyme SspI to distinguish Sq-8/NFA-8 lines containing *mini1a* using a SNP at position 13,405,811 of Chr1, as well as corresponding *mini1a* junction PCR of *mini1a* at the expected breakpoint junction site

Previously, we produced haploids by crossing the haploid inducer GFP-tailswap as female parent with the F1 hybrid of *Arabidopsis thaliana* ecotypes Sq-8 and NFA-8 as male to generate instantaneous doubled haploid inbred lines (Seymour et al. 2012). We surveyed the putative dihaploids by skim sequencing and single nucleotide polymorphism (SNP) genotyping specific to the maternal Col-0 genome. The majority of progeny were pure dihaploids, which lack any maternal SNP (Fig. 1). In addition to a few hybrids that escaped the screen (Seymour et al. 2012; Ravi et al. 2014), two individuals displayed Col-0 SNP specifically in the centromeric region of a single chromosome, in which two are described here. Based on SNP analyses, all five full-length haploid chromosomes are derived from the parental ecotypes of the F1 paternal parent but also appear to carry an additional copy of a truncated chromosome from Col-0, which is the maternal haploid inducer ecotype (Fig. 2A, B).

**Fig. 3:**
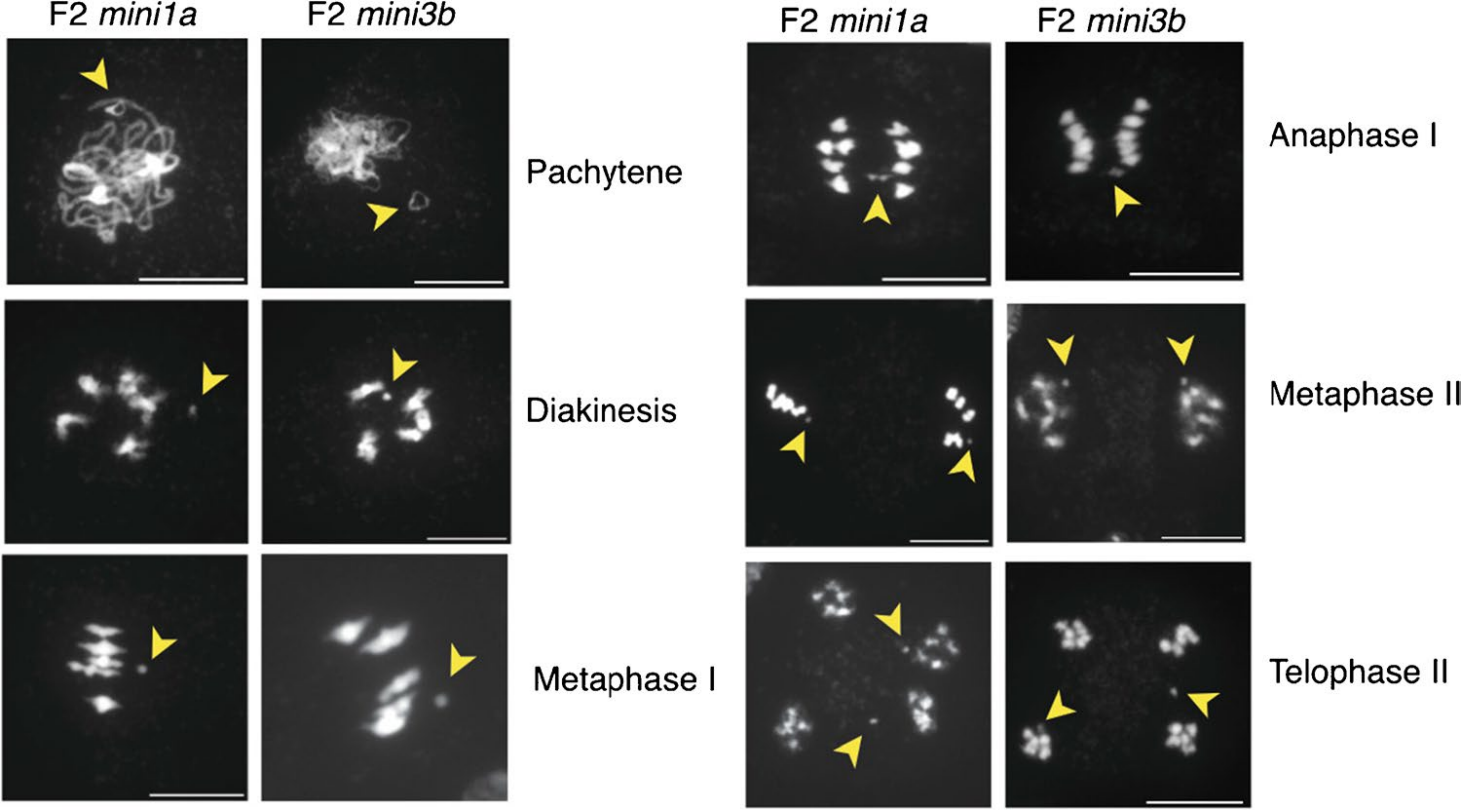
Meiotic behavior of male meiocytes of *mini1a* and *mini3b* in F2 doubled haploid lines. Yellow arrowheads indicate presumed location of minis during meiotic stages. Scale bar = 10 μm

We characterized two of these lines, which had spontaneously gained diploidy (Ravi et al. 2014), by high throughput sequencing. Read dosage analysis demonstrated the Col-0 DNA signal is associated with the presence of a deletion derivative spanning the centromeric region, which appears as a partial third copy (Fig. 2C, D). We called these, respectively, *mini1a* and *mini3b*. In pachytene spreads, *mini1a* and *mini3b* appeared as small circular chromosomes displaying intense DAPI staining at the centromeric regions (Fig. 3).

We focused further on *mini1a* (Fig. 2E). Using a modified breakpoint assembly method that leverages read pairs (Tan et al. 2015), we identified the junction that circularized the chromosome. Employing flanking primers (Mini1a), we performed PCR followed by Sanger sequencing. Analysis of the amplified junction region structure showed a 17-bp duplication of Chr1 regions near the major breakpoint at position 17,824,627. According to the TAIR10 reference, *mini1a* is 4486 kb in size. Considering the full centromere assembly, however, its predicted size is 6598 kb (Naish et al. 2021). The same approach was unsuccessful in generating the breakpoint junction for *mini3b*, although data from our cytological observations (Fig. 3) suggest a similar, circular breakpoint junction for *mini3b*.

Because we have the reconstructed breakpoint junction for *mini1a* and were able to accurately estimate the exact chromosomal position, we scanned the Araport11 sequence annotation spanning the *mini1a* region and found that *mini1a* contains 386 predicted genes and thousands of transposons, as expected from a chromosomal element formed by the centromere and the adjacent pericentromeric regions.

### Parental transmission

Both *mini1a* and *mini3b* were transmitted for at least 6 selfing generations, although accurate transmission efficiency could not be evaluated. To determine the meiotic transmission of *mini1a* and its stability over multiple generations, the line carrying *mini1a* was selfed or hybridized with Landsberg *erecta* (L*er*) (Fig. 1). The *mini1a*-specific junction provided convenient genotyping via PCR markers (Fig. 2F). Efficiency during selfing ranged from 22 to 47% (Table 2). Interestingly, in generations S7, S8, and S10, three progeny families did not inherit *mini1a* (Table 2), suggesting occasional instability and loss from the germ line. Using reciprocal crosses to wild-type (WT) L*er gl-1*, the transmission rates of *mini1a* through the female and male were 12.5 and 10.7%, respectively (Table 3). The observed transmission rate upon selfing is therefore consistent with the combined probability of male and female transmission (0.218; see Methods), a number consistent with the measured rate. Taken together, these results indicated that minichromosomes produced by haploid induction can be transmitted at a rate consistent with those of trisomic chromosomes (Koornneef and Van der Veen 1983).

**Table 2:** Transmission rates of the *mini1a* in selfed progenies over eleven generations

Generation mini1a*	No. of families (Individuals) assayed	No. of individuals carrying minis	Transmission rate average, %	Transmission rate range, %
F2	1 (17)	8	47	na
S1	1 (10)	6	60	na
S2	1 (10)	3	30	na
S3	1 (7)	3	43	na
S4	1 (15)	6	40	na
S5	2 (162)	76	47	46–48
S6	5 (299)	128	43	31–52
S7	11 (664)	173	23	0–55
S8	8 (408)	122	29	0–56
S9	16 (789)	213	26	6–48
S10	4 (250)	28	19	0–39

*Pool data per combination; *na*, not applicable

**Table 3:** Parental transmission rate of the *mini1a*

Parental combination*	No. of individuals scored	No. of individuals carrying minis	Transmission rate %
*mini1a* ✕ WT	320	40	12.5
WT ✕ *mini1a*	168	18	10.7

*WT*, *Landsberg erecta* glabra; *pool data per combination

### Cytological behavior

**Fig. 4:**
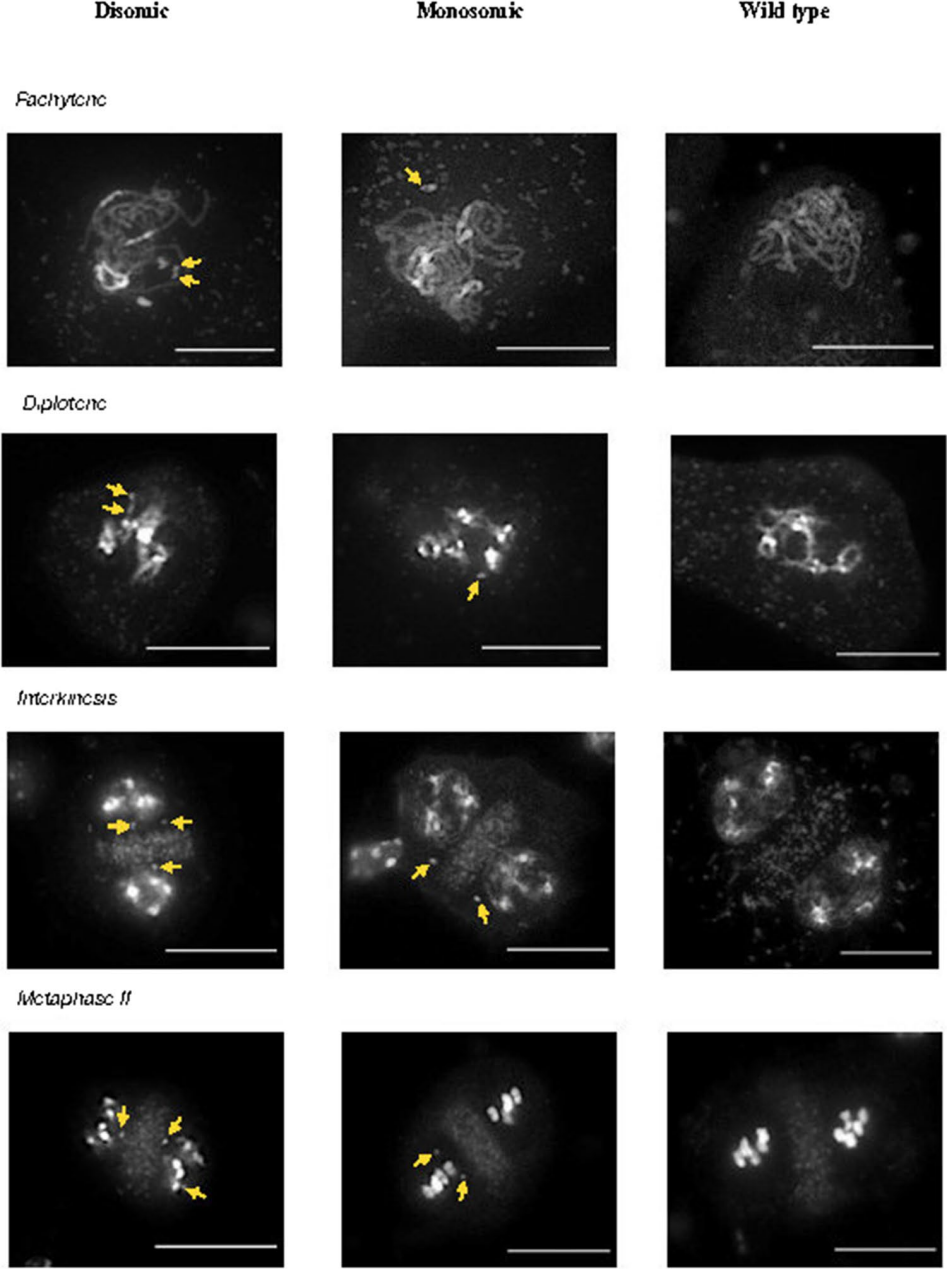
Meiotic behavior of male meiocytes from monosomic and disomic *mini1a* in S6 lines, and a wild type. Yellow arrowheads indicate presumed location of minis during meiotic stages. Scale bar = 10 μm

We observed the behavior of *mini1a* and *mini3a* in the F2 dihaploids (Fig. 3) and for *mini1a* in the following generations (Fig. 4). At pachytene I of meiosis, both *mini1a* and *mini3a* form distinct, unpaired circles with a pericentromeric knob (Fig. 3). The single, unpaired state is supported by the appearance of separate and punctate structures at diakinesis 1 (Fig. 3). The offset position from the metaphase plate apparent at metaphase I suggests attachment to a single pole. At anaphase I, however, *mini1a* can lag and can appear under tension, suggesting merotelic attachment, possibly leading to premature separation of the sister chromatids. In the S6 generation, *mini1a* appeared as a disomic or a monosomic particle (Fig. 4) and displayed behavior similar to that observed in the F2 generation. Cytology indicated that a predominantly single-copy *mini1a* could also exist in a disomic state, perhaps because of early sister chromatid separation. When monosomic, sister chromatids could behave normally and separate at anaphase II.

### Targeted production of minichromosomes from chromosome 4

**Fig. 5:**
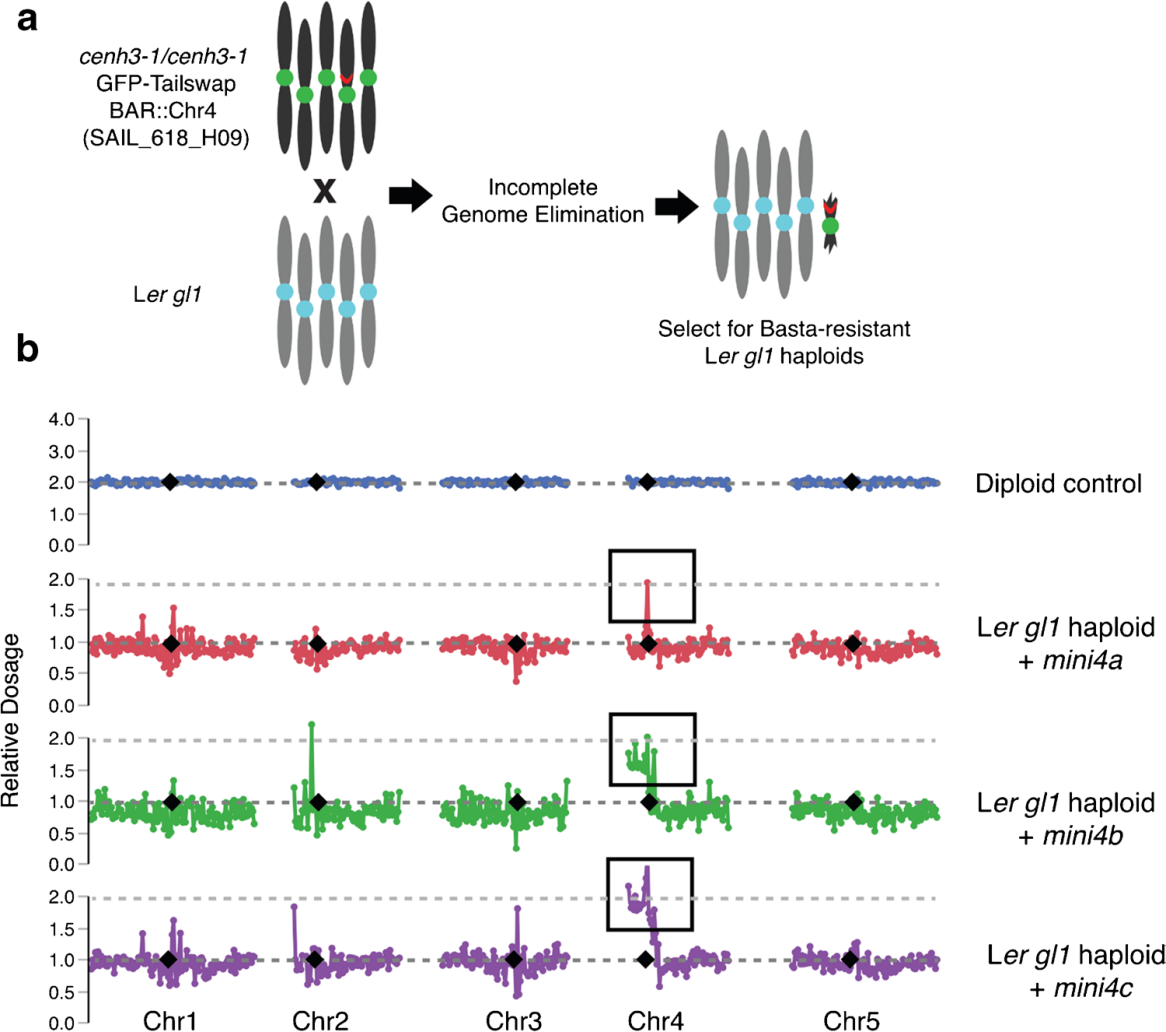
Derivation of selectable Basta-resistant minichromosomes from chromosome 4 (Col-0 ecotype) in the Landsberg *erecta* (L*er*) background. **a** Schematic representation of haploid induction cross used to generate selectable minichromosomes from Chr 4 of *Arabidopsis*. **b** Dosage plots of three L*er gl1* haploids carrying Chr4 minichromosomes carrying selectable marker bar, are shown here. On the *y*-axis, the relative dosage is set at 2 for the main signal for wild-type diploid control and at 1 for the haploids. Outside the highlighted regions, occasional singleton signals can be observed. This is a common finding for centromeric and heterochromatic regions of control individuals and are not considered relevant genomic variation

Spurred by the serendipitous discovery of *mini1a* and *mini3b*, we tested whether selection for herbicide resistance in a pericentric locus could facilitate rapid and reliable isolation of minichromosomes during haploid induction. To test this, we chose a Basta-resistant T-DNA line, SAIL_618_H09 (Col-0 ecotype), in which the location of the T-DNA was mapped to the pericentromeric region of chromosome 4, and introgressed this pericentromeric T-DNA into a GFP-tailswap haploid inducer (HI). Using a HI line that contains the T-DNA, we performed haploid induction crosses with L*er gl1* as the male (Fig. 5). Several hundred trichomes F1 L*er gl1* haploids were transplanted on soil. After applying Basta herbicide on 3-week-old plants, we isolated 3 Ler *gl1* haploids that were Basta resistant. Sequencing of these F1 lines showed that two of the additional chromosomes consisted of CEN4 plus the short arm. The third minichromosome, *mini4a*, was considerably smaller. In all cases, the dosage plot analysis revealed a twofold increase in the centromeric regions, consistent with disomy in a monoploid genome. Taken together, our results demonstrate that positive selection for a marker integrated either in the centromeric or pericentromeric region of a chromosome can aid rapid selection of stable minichromosomes, bypassing the tedious cytological analysis and expensive high-throughput sequencing screen to identify plants containing minichromosome(s). Furthermore, minichromosomes can express transgenic markers.

## Discussion

We have discovered that 1–2% of phenotypically normal arabidopsis haploids resulting from crosses to the CENH3-based GFP-tailswap haploid inducer carry minichromosomes (minis) derived from centromeric and adjacent pericentromeric regions. We detected the minis by cytological methods, genotyping, and dosage profiling genomic reads. We demonstrated that the minis originate from the haploid inducer genome using sequence analysis. We found that *mini1a* is circular with a junction between two sites that flank the centromere of Chr1, which was confirmed by cytological methods. In addition, a mechanism for generating minichromosomes for specific chromosomes was developed, and we showed that minis could be identified easily when the haploid inducer carried a selectable marker in the centromeric region of Chr4. These elements ranged between 3 and 10 Mb in size. The line carrying *mini1a* appeared as a single unpaired circle with a distinct DAPI-stained knob at prophase and metaphase (Fig. 3). Occasionally, two unpaired circles per cell were visible. In self crosses, *mini1a* was transmitted to ~ 25% of the progeny, which is consistent with the observed ~ 0.12 transmission rate through male or female gametes. Trisomics of Chr1 display very similar transmission rates upon selfing (Koornneef and Van der Veen 1983). This similar efficiency, however, is coincidental because, compared to trisomics of Chr1, *mini1a* displayed lower female transmission and higher male transmission. Considering the parental transmission rates, the predicted frequency of inheriting two copies of *mini1a* is 0.125 * 0.107 = 0.013, a relatively infrequent event. For certain plants, we observed a very low transmission (Fig. 1), which may be explained by mitotic instability and loss of the minichromosome. Murata et al. (2006) described an increase in transmission when their Ler-derived Chr4 mini (referred to as *mini4S* henceforth) was backcrossed into the Col-0 ecotype. This suggested a genotypic background effect on the transmission of a minichromosome. In addition, similar to our *Bar*-derived *mini4b* and *mini4c*, Murata’s *mini4S* contains the entire short arm of Chr4, which is known to carry the ribosomal RNA genes and function as a nucleolus organizer region (NOR). The presence of telomeres may favor associations of NORs during meiosis (Murata et al. 2006), which could lead to different outcomes when compared to circularized minis such as *mini1a* and *mini3b* that are derived from isocentric chromosomes that do not contain NORs.

### Minis from genomic instability

**Fig. 6:**
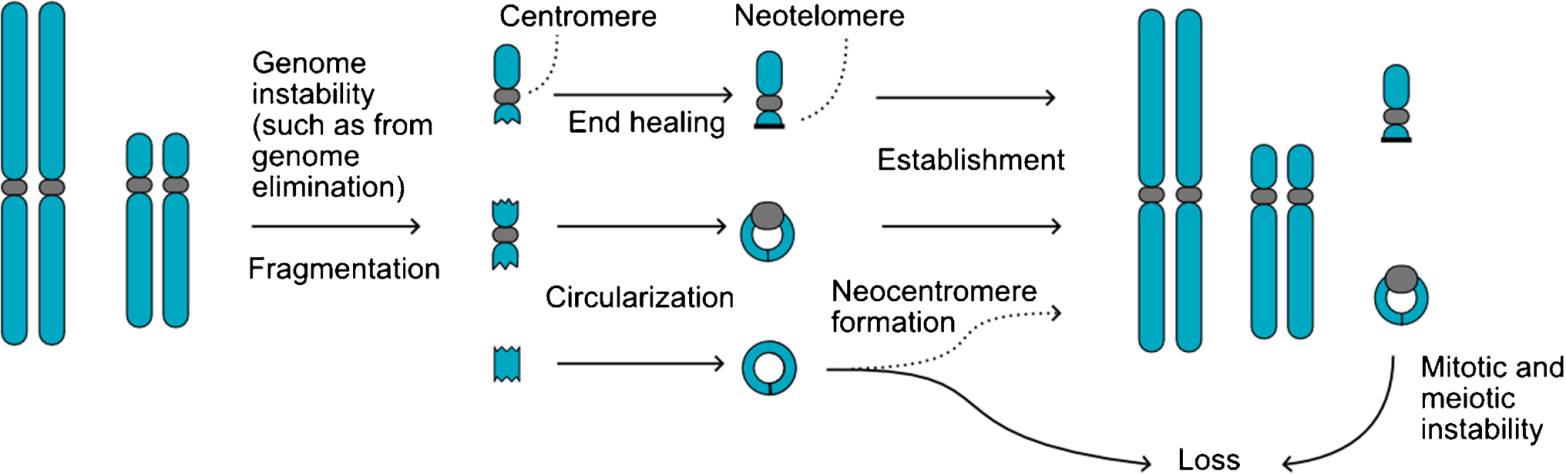
Formation of minichromosomes as a result of genome instability. We propose that minichromosomes arise from genome instability leading to fragmentation. Chromosomal fragments that contain a centromere or form a neocentromere can be stabilized by either formation of telomeres or by circularization. The resulting chromosomes are typically unstable due to their small size or circularity

In CENH3-mediated haploid induction, about ${~}^{1}\!\left/ \!{~}_{3}\right.$ of the progeny are aneuploid, and about a ${~}^{1}\!\left/ \!{~}_{3}\right.$ of these aneuploids carry chromosomes rearranged by chromoanagenesis (Comai and Tan 2019). Typically, aneuploids carrying these shuffled chromosomes are developmentally abnormal and sterile, probably due to imbalance of many genes and possibly to the action of novel gene fusions (Tan et al. 2015). We propose that minis arise when a mis-segregated chromosome is captured in a micronucleus, and after fragmentation, a chromosomal segment carrying the centromeric region circularizes and is restituted to the nucleus (Fig. 2E). Potentially, formation of telomeres may also stabilize certain centromeric fragments. Instability is commonly associated with haploid induction (Maheshwari et al. 2015; Tan et al. 2015; Kuppu et al. 2015; Amundson et al. 2021; Sun et al. 2022) and with wide crosses (Madlung et al. 2005; Shibata et al. 2013), and minis are likely to arise in these crosses (Seymour et al. 2012; Tan et al. 2015; Kuppu et al. 2015). One question is whether minichromosomes require mutations in specialized loci of DNA metabolism for their formation and maintenance. The frequencies observed are inconsistent with those expected for mutations, even if multiple loci could be involved. Furthermore, transmission in outcrosses (Table 3) indicates that no recessive mutation is required. In many instances, minichromosomes may not be associated with detectable traits, likely because of the reduced number of genes that are imbalanced. We have no direct evidence of gene expression for *mini1a*, but successful selection for mini4a, 4b, and 4c demonstrates that the transgenic marker is expressed. Identification through genomic analysis is also challenging in most species because of the difficulty in detecting CNV in the background of complex, variable, and uncharacterized centromeric regions (Hardigan et al. 2016; Hufford et al. 2021). Nonetheless, reduced-size, additional chromosomes can be found both by sequencing (Shibata et al. 2013; Amundson et al. 2021) or by cytological analysis (Shibata et al. 2013). The mechanisms by which minis can originate are illustrated in Fig. 6. Linear or circularized minis that retain centromeres during periods of genome instability are capable of germline transmission and maintenance as extrachromosomal entities.

Chromosome fragments that contain the centromere can either heal their exposed ends or circularize. Acentric fragments can persist through a number of divisions but are eventually lost unless they form a neocentromere. Linear or circular minichromosomes with functional centromeres can persist through meiotic generations, but their redundancy and suboptimal structure will eventually result in loss.

### Method for isolation of minichromosomes

We demonstrate that minis can be identified readily when a haploid inducer carries a marker in the pericentromeric region (Fig. 5). Among the phenotypically normal haploids, those expressing the marker typically carry a novel mini. The formation of circular vs. linear minichromosomes may depend on local context and features. The method is applicable to any species where instability results from haploid induction and provides an approach toward studying and exploiting minis. Mini’s have been described for nearly 100 years, starting with the pioneering work of McClintock on maize circular chromosomes (McClintock 1932). Their potential for vectoring stacks of valuable genes and avoiding linkage drag has drawn considerable attention. One challenge is the loading of genes on the mini, for which multiple solutions based on transformation and induced recombination have been proposed (Birchler 2015; Kumar et al. 2016; Anand et al. 2019; Dong and Ronald 2021). Our experiments indicate that dominant markers in the centromeric region suggest an additional approach: minis carrying valuable genes could be generated if these genes are located in pericentromeric regions. It is also possible that minis combining distal segments of euchromatin with a centromeric segment could be selected in vivo if these genes have been previously marked with a suitable transgene.

### Remaining challenges and opportunities

Specific information on the mitotic and meiotic instability of a mini, while not a specific objective of this study, should be obtained before its use on a commercial scale. Mitotic instability would affect the use of a minichromosome vector by resulting in chimeric plants. During mitosis, an uneven number of crossovers between sister chromatids can fuse replicated circular chromosomes and initiate cycles of breakage-fusion-bridge events that result in loss or duplication of DNA as well as missegregation. Another malfunction that can increase missegregation is early loss of sister chromatid cohesion.

Instability is also likely in meiosis (Han et al. 2007). The type of mini characterized here is derived from the normal chromosome complement and, therefore, it resembles trisomy. During meiosis, trisomy is a naturally unstable state when a full-length chromosome is involved (Koornneef and Van der Veen 1983). In addition, our minis do not appear to pair efficiently with their full-size homologs. The small size of *mini1a*, its reduced content of euchromatin, and the lack of telomeres likely contribute to this property. Lack of homologous pairing causes premature mobilization by the spindle, leading to increased missegregation. Instability during meiosis may be less damaging to vector utilization. Lack of pairing, even when two minis are present, destabilizes minis at metaphase I of meiosis and likely contributes to reduced transmission in gametes.

Both meiotic and mitotic instability may be overcome if the mini includes a selectable marker gene (Han et al. 2007). Mitotic chimerism could be overcome if the mini carries a gene essential for cellular proliferation. Incomplete meiotic transmission could be ameliorated by the presence of a detectable marker, although it would increase seed production costs. Notably, for certain uses, instability is not problematic but useful. For example, provision of genome editing transgenes on a mini could be advantageous: after genome editing, transgene-free progeny could be easily identified.

## Conclusion

In conclusion, the frequent formation of mini chromosomes during haploid induction and as a byproduct of genome elimination provides a natural context during which karyotypic novelty arises. This property can now be exploited for both basic and applied studies.

## Data Availability

Raw sequencing reads from this project can be accessed via BioProject ID PRJNA826082. The *mini1a* lines are available from LC, *mini3b*, *mini4b*, and *mini4c* from EHT.
